# Lobar microbleeds are associated with cognitive impairment in patients with lacunar infarction

**DOI:** 10.1038/s41598-020-73404-6

**Published:** 2020-10-02

**Authors:** Masahiro Nakamori, Naohisa Hosomi, Keisuke Tachiyama, Teppei Kamimura, Hayato Matsushima, Yuki Hayashi, Eiji Imamura, Shinichi Wakabayashi, Hirofumi Maruyama

**Affiliations:** 1 Department of Clinical Neuroscience and Therapeutics, Hiroshima University Graduate School of Biomedical and Health Sciences, Hiroshima, Japan; 2 Department of Neurology, Suiseikai Kajikawa Hospital, Hiroshima, Japan; 3 Department of Neurology, Chikamori Hospital, 1-1-16 Ohkawasuji, Kochi, 780-8522 Japan; 4 Department of Disease Model, Research Institute of Radiation Biology and Medicine, Hiroshima University, Hiroshima, Japan; 5 Department of Neurosurgery, Suiseikai Kajikawa Hospital, Hiroshima, Japan

**Keywords:** Neurology, Risk factors

## Abstract

Associations between cognitive decline and cerebral microbleeds (CMBs) have received increasing attention. An association between CMB distribution (deep or lobar) and cognitive decline has been reported, but these findings are controversial. We investigated the association between magnetic resonance imaging (MRI) findings, including CMBs, and cognitive function in patients with first-ever lacunar infarction. We retrospectively included consecutive patients admitted with first-ever lacunar infarction identified by MRI from July 1, 2011, to December 31, 2018. We excluded patients diagnosed with dementia, including strategic single-infarct dementia, before or after the onset of stroke. The Mini-Mental State Examination (MMSE) was performed within 3 days of admission. We searched the records of 273 patients (age 72.0 ± 11.2 years, 95 females). The median MMSE score was 27 (interquartile range 25.5–29). In a univariate analysis, the MMSE score was associated with age, body mass index (BMI), education, dyslipidemia, chronic kidney disease (CKD), periventricular hyperintensity, medial temporal atrophy, lobar CMBs, and mixed CMBs (p < 0.20). The lacunar infarction location was not associated with the MMSE score. In a multivariate analysis of these factors, lobar CMBs (p < 0.001) and mixed CMBs (p = 0.008) were independently associated with the MMSE score. Lobar CMBs were associated with cognitive impairment.

## Introduction

Cognitive impairment, which leads to morbidity and mortality, is one of the most critical issues in the field of population health, healthcare, and medical economics^1,2^. While aging is a risk factor for cognitive impairment, cerebral small vessel diseases (cSVDs) have also attracted attention for their roles in this condition^3–5^. White matter lesions (WMLs), lacunar infarcts, and cerebral microbleeds (CMBs) on magnetic resonance imaging (MRI) are hallmarks of cSVDs^6–8^.

The CMBs on gradient-echo T2*-weighted imaging (T2*WI), which are characterized histologically by the presence of hemosiderin around small vessels, are now accepted as a manifestation of cerebral small vessel pathologies, including hypertensive small vessel disease and cerebral amyloid angiopathy (CAA)^9,10^. Currently, the pathological differences in CMBs according to distribution are well known. The CMBs in deep regions (deep CMBs) are associated with hypertensive microangiopathy, whereas lobar CMBs share risk factors with CAA^11,12^.

Increasing attention has been paid to associations between cognitive impairment and CMBs^13^. CMBs are often detected in individuals with dementia, such as patients with Alzheimer’s disease (AD)^14^, vascular cognitive impairment^15^, or memory loss^16^. Several studies have shown an association between CMBs and cognitive impairment^17,18^. An association between the distribution of CMBs (deep or lobar) and cognitive impairment has been reported, but the results are still controversial^19–23^.

In this study, we investigated the association between MRI findings, including CMBs, and cognitive function in patients with a first-ever acute stroke diagnosed with lacunar infarction.

## Materials and methods

### Ethics

The study protocols were approved by the ethics committee of Suiseikai Kajikawa Hospital (approval number 2019-07) and performed in accordance with the national government guidelines based on the 1964 Declaration of Helsinki. The ethics committee of Suiseikai Kajikawa Hospital waived the requirement for written informed consent for this study owing to its retrospective nature; upon admission, the included patients agreed that their data could be used for future studies.

### Data availability statement

The data that support the findings of this study are available from the corresponding author on reasonable request.

### Subjects

We retrospectively included consecutive patients admitted with a first-ever acute stroke diagnosed with lacunar infarction via MRI from July 1, 2011, to December 31, 2018. Lacunar infarction was determined according to the criteria from the Trial of Org 10,172 in Acute Stroke Treatment^24^. We excluded patients who had been diagnosed with dementia, including strategic single infarct dementia, before or after the onset of stroke. Dementia was diagnosed using the 10th revision of the International Statistical Classification of Diseases and Related Health Problems. The diagnosis was confirmed by two stroke neurologists (HM and EI).

### Data acquisition

The Mini-Mental State Examination (MMSE) was performed within 3 days of admission by a clinical technologist specialized in psychology. We collected data from hospital records.

Baseline clinical characteristics, including age, sex, body mass index (BMI), years of education, drinking and smoking habits, and comorbidities [hypertension, diabetes mellitus, dyslipidemia, chronic kidney disease (CKD)], were collected from all patients. Comorbidities were defied as previously reported^25^. Hypertension was defined as a confirmed blood pressure of 140/90 mm Hg or higher at rest at 1 week after stroke onset or the use of antihypertensive medicine before admission. Diabetes mellitus was defined as a glycated hemoglobin level of ≥ 6.5%, fasting blood glucose level of ≥ 126 mg/dl, or the use of antidiabetic medication. Dyslipidemia was defined as fulfillment of any of the following criteria: total cholesterol level of ≥ 220 mg/dl, low-density lipoprotein cholesterol level of ≥ 140 mg/dl, high-density lipoprotein cholesterol level of < 40 mg/dl, triglyceride level of ≥ 150 mg/dl, or use of antihyperlipidemic medication. Renal functioning was calculated with the estimated glomerular filtration rate (eGFR) using a revised equation for the Japanese population as follows: eGFR (ml min^−1^ 1.73 m^−2^) = 194 × (serum creatinine)^−10094^ × (age)^−0.287^ × 0.739 (for women)^26^. CKD was defined as an eGFR < 60 ml min^−1^ 1.73 m^−2^. There were no patients with atrial fibrillation in this study.

### Brain MRI

The MRI was performed with a 1.5 T scanner (Avanto, Siemens Medical Systems, Erlangen, Germany) or a 3.0 T scanner (Spectra, Siemens Medical Systems, Erlangen, Germany). The imaging protocol consisted of fluid-attenuated inversion recovery (FLAIR) imaging (TR = 12,000 ms and TE = 87 ms for turbo spin echo, inversion time = 2800 ms, FOV = 22 cm, matrix size = 226 × 384, slice thickness = 5.0 mm, interslice spacing = 1.5 mm) and T2*-weighted imaging (TR = 617 ms and TE = 14 ms for gradient echo, FOV = 22 cm, matrix size = 224 × 320, slice thickness = 5.0 mm, interslice spacing = 1.5 mm). The severity of the WMLs [deep and subcortical white matter hyperintensity (DSWMH) and periventricular hyperintensity (PVH)] was rated visually from the FLAIR images using the Fazekas scale (DSWMH: grade 1, punctuate; grade 2, early confluence; and grade 3, confluent; and PVH: grade 1, caps or lining; grade 2, bands; and grade 3, irregular extension into the deep white matter)^27^. The patients with PVH/DSWMH of grades 0 to 1 were assigned to the mild group, and those with PVH/DSWMH of grades 2 to 3 were assigned to the severe WML group. The degree of medial temporal atrophy was semiquantitatively evaluated as described in previous reports^28–30^, using a 5-point score scale ranging from 0 (no atrophy) to 4 (severe atrophy). The presence and number of CMBs were evaluated from T2*WI. CMBs were defined as homogeneous round lesions measuring 2 to 10 mm in diameter and characterized by signal intensity loss on gradient-echo MRI^31,32^. The locations of the CMBs were classified as follows: strict deep (basal ganglia, thalamus, brain stem and cerebellum), strict lobar (cortex, subcortex and white matter), and mixed (both lobar and deep). Two stroke neurologists (MN and KT) who were unaware of the clinical details of the patients graded the severity of the WMLs and medial temporal atrophy and counted the CMBs. Each interrater kappa value for DSWMH grade (mild vs severe), PVH grade (mild vs severe), medial temporal atrophy grade (0–4), deep CMB presence/number, lobar CMB presence/number, and mixed CMB presence/number was within the range of 0.75 to 0.85. Each intrarater kappa value for DSWMH grade (mild vs severe), PVH grade (mild vs severe), the grade (0–4) of medial temporal atrophy, deep CMB presence/number, lobar CMB presence/number, and mixed CMB presence/number was within the range of 0.80 to 0.90.

### Statistical analysis

The data are expressed as the mean ± standard deviation or the median [25% indicates interquartile range (IQR)–75% IQR] for continuous variables, and frequencies and percentages for discrete variables. Statistical analysis was performed using JMP 14 statistical software (SAS Institute, Inc., Cary, NC, USA). The statistical significance of the intergroup differences was assessed using unpaired *t-*tests, Mann–Whitney *U* tests, or χ^2^ tests as appropriate. We divided the patients into four groups: CMB-negative, CMB-deep, CMB-lobar, and CMB-mixed. Then, the data were analyzed with Steel tests. To determine the association with the MMSE score, a univariate analysis was performed, and p = 0.20 was used as the cutoff level. Then, a multifactorial least-squares linear regression analysis was performed with selected factors that were identified from the univariate analysis. We considered p < 0.05 to be significant.

## Results

**Figure 1:**
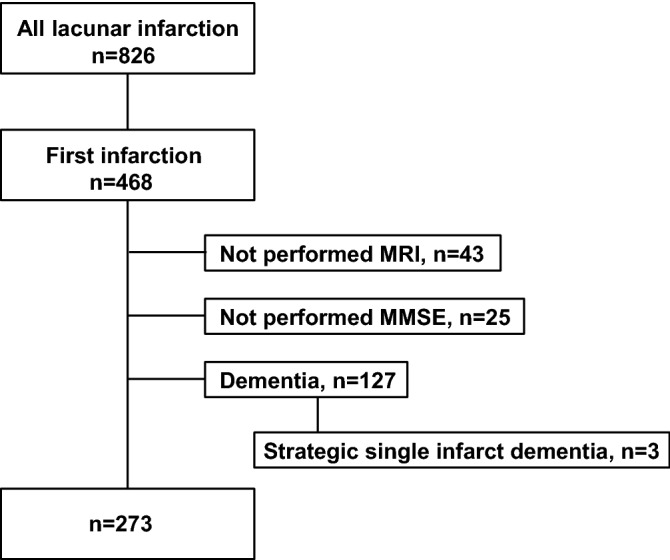
Flow chart of the inclusion and exclusion criteria. MRI, magnetic resonance imaging; MMSE, Mini-Mental State Examination.

**Figure 2:**
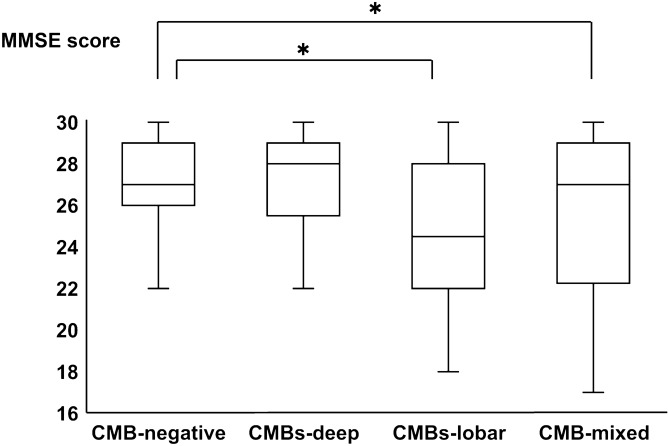
Comparison of the MMSE scores among the CMB-negative, CMB-deep, CMB-lobar, and CMB-mixed groups. The MMSE scores of the CMB-lobar and CMB-mixed groups were significantly lower than those of the CMB-negative group. Additionally, the MMSE score of the CMB-lobar group was lowest among all of the groups. MMSE, Mini-Mental State Examination; CMBs, cerebral microbleeds.

A total of 826 patients were diagnosed with lacunar infarction, and 468 patients were first-ever stroke patients. Among these patients, 43 patients without MRI, 25 patients without MMSE, and 127 patients diagnosed with dementia, including strategic single infarct dementia (n = 3), before or after the onset of stroke were excluded. Ultimately, we analyzed 273 stroke patients (age 72.0 ± 11.2 years, 95 females; Fig. 1). None of the patients showed alterations in consciousness. We did not add any new psychiatric drugs, including sleeping pills, in the 3 days from admission to the MMSE. The median MMSE score was 27 (25.5 to 29, 25% IQR–75% IQR) (Fig. 1). The patients’ backgrounds are shown in Table 1. CMBs were detected in 73 of the 273 (26.7%) patients. The numbers of patients with deep CMBs, lobar CMBs, and mixed CMBs were 37, 12, and 24, respectively. Among the 73 CMB-positive patients, the mean numbers of deep CMBs and lobar CMBs were 6.58 ± 12.75, 3.56 ± 6.88, respectively. We divided the patients into four groups: CMB-negative, CMB-deep, CMB-lobar, and CMB-mixed. The MMSE score of each group is shown in Fig. 2. Steel tests revealed that the MMSE scores of the CMB-lobar and CMB-mixed groups were significantly lower than those of the CMB-negative group (p < 0.05).

**Table 1:** Patient background information.

	Total (n = 273)	CMB-positive (n = 73)	CMB-negative (n = 200)	P value
Age, year	72.0 ± 11.2	72.2 ± 9.2	72.0 ± 11.28	0.849
Sex (female), n (%)	95 (34.8)	19 (26.0)	76 (38.0)	0.066
Body mass index, kg/m2	23.8 ± 3.5	23.6 ± 3.1	23.8 ± 3.7	0.735
Education, years	12.3 ± 2.4	12.6 ± 2.4	11.5 ± 2.3	0.001
MMSE score, median (IQR)	27 (25.5–29)	27 (24–29)	27 (26–29)	0.003
Hypertension, n (%)	206 (75.5)	64 (87.7)	142 (71.0)	0.005
Diabetes mellitus, n (%)	69 (25.3)	16 (21.9)	53 (26.5)	0.441
Dyslipidemia, n (%)	151 (55.3)	34 (46.6)	117 (58.5)	0.079
Chronic kidney disease, n (%)	82 (30.0)	28 (38.4)	54 (27.0)	0.070
Current smoker, n (%)	102 (37.4)	27 (37.0)	75 (37.5)	0.938
Habitual drinker, n (%)	109 (39.9)	31 (43.1)	78 (39.0)	0.547
**Location of infarction**				
Corona radiata, n (%)	86 (31.5)	28 (38.4)	58 (29.0)	0.141
Basal ganglia, n (%)	19 (7.0)	5 (6.8)	14 (7.0)	0.966
Capsulae internae, n (%)	56 (20.5)	14 (19.2)	42 (21.0)	0.741
Thalamus, n (%)	65 (23.8)	12 (16.4)	53 (26.5)	0.084
Brain stem, n (%)	47 (17.2)	14 (19.2)	33 (16.5)	0.604
**MRI findings**				
DSWMH severe, n (%)	150 (54.9)	48 (65.8)	102 (51.0)	0.030
PVH severe, n (%)	166 (60.8)	55 (75.3)	111 (55.5)	0.030
Medial temporal atrophy (IQR)	1 (0–2)	1 (0–2)	1 (0–2)	0.353
**CMBs**				
Deep, n (%)	37 (13.6)			
Lobar, n (%)	12 (4.4)			
Mixed, n (%)	24 (8.8)			

CMBs, cerebral microbleeds; MMSE, Mini-Mental Scale Examination; IQR, interquartile range; MRI, magnetic resonance imaging; DSWMH, deep and subcortical white matter hyperintensity; PVH, periventricular hyperintensity. Data are presented as the mean ± standard deviation, median (25% IQR–75% IQR), or number of patients (%). * indicates < 0.05.

We divided the patients into a lobar-CMB-positive group (CMB-lobar and CMB-mixed) and a lobar-CMB-negative group (CMB-negative and CMB-deep). Two groups were compared with the backgrounds shown in Table 1. There were no significant differences between the two groups except for the MMSE score and CKD (Supplemental Table 1).

Next, we investigated the associations with the MMSE score using the factors listed in Table 1. On univariate analysis, the MMSE score was associated with age, BMI, education, dyslipidemia, CKD, PVH, medial temporal atrophy, lobar CMBs, and mixed CMBs (p < 0.20). The location of the lacunar infarction was not associated with the MMSE score. We performed a multivariate analysis with these factors and found that age, BMI, medial temporal atrophy, lobar CMBs (p < 0.001), and mixed CMBs (p = 0.008) were independently associated with the MMSE score (Table 2). In addition, instead of the type of CMBs, we used the number of deep or lobar CMBs and investigated associations with the MMSE score using the factors listed in Table 1. The multivariate analysis revealed that age, medial temporal atrophy, and the number of lobar CMBs (p = 0.001) were independently associated with the MMSE score (Supplemental Table 2). Three patients were diagnosed with strategic single infarct dementia. Although these patients were included in the analysis, the association between the MMSE and background factors did not change.

**Table 2:** Associations between multiple factors and decreases in MMSE scores.

	Univariate analysis	Multivariate analysis					
	P value	Predictive value	P value	VIF	Predictive value	P value	VIF
Age	< 0.001	− 0.061	0.004	2.78	− 0.064	0.003	2.77
Sex (female)	0.392						
Body mass index	0.001	0.095	0.026	1.13	0.093	0.031	1.13
Education	0.001	0.013	0.853	1.43	0.008	0.915	1.43
Hypertension	0.353						
Diabetes mellitus	0.214						
Dyslipidemia	0.113	0.018	0.903	1.08	0.026	0.859	1.08
Chronic kidney disease	0.002	− 0.276	0.088	1.10	− 0.300	0.067	1.09
Current smoker	0.494						
Habitual drinker	0.221						
**Location of infarction**							
corona radiata	0.826						
basal ganglia	0.608						
capsulae internae	0.718						
thalamus	0.259						
brain stem	0.335						
**MRI findings**							
DWMH severe	0.304						
PVH severe	0.056	− 0.006	0.968	1.19	− 0.006	0.969	1.20
Medial temporal atrophy	< 0.001	− 1.491	< 0.001	2.74	− 1.537	< 0.001	2.73
CMBs (deep)	0.686						
CMBs (lobar)	0.004	− 0.811	< 0.001	1.05			
CMBs (mixed)	0.002				− 0.695	0.008	1.05

MMSE, Mini-Mental Scale Examination; VIF, variance inflation factor; MRI, magnetic resonance imaging; DSWMH, deep and subcortical white matter hyperintensity; PVH, periventricular hyperintensity; CMBs, cerebral microbleeds. * indicates < 0.05.

We analyzed the subscores of the MMSE scores among the four groups (Table 3). There was no significant difference between the CMB-deep and CMB-negative groups. However, the subscores for orientation and visuospatial function were significantly lower in the CMB-lobar group than in the CMB-negative group after adjustment for BMI and CKD (p = 0.004 and 0.009, respectively). In addition, the subscores for orientation (p = 0.002) and visuospatial function (p = 0.039) were significantly lower in the CMB-mixed group than in the CMB-negative group after adjustment for BMI and CKD (p = 0.002 and 0.039, respectively).

**Table 3:** Comparisons of MMSE subscores.

	CMBs-negative	CMBs-deep	CMBs-lobar	CMBs-mixed
Orientation	10 (9–10)	10 (9–10)	9 (7.25–10)*	9.5 (8–10)*
Immediate recall	3 (3–3)	3 (3–3)	3 (3–3)	3 (3–3)
Attention and calculation	5 (2–5)	5 (2–5)	3 (1.25–5)	3.5 (2–5)
Delayed recall	2 (2–3)	2 (2–3)	2 (1.25–3)	2 (1–3)
Language	9 (9–9)	9 (8–9)	9 (7–9)	9 (8–9)
Visuospatial cognition	1 (1–1)	1 (1–1)	1 (0.25–1)*	1 (1–1)*

MMSE, Mini-Mental State Examination; CMBs, cerebral microbleeds. Data are presented as the median [25% interquartile range (IQR)–75% IQR].

*A significant difference was observed compared with the CMBs-negative group (p < 0.05).

## Discussion

In this study, we focused on patients with a first-ever lacunar infarction who were not diagnosed with dementia, including strategic single infarct dementia, and investigated the association between the MMSE score and the CMBs. This investigation revealed that the MMSE score is associated with lobar CMBs. This finding suggests that lobar CMBs are important factors for cognitive impairment.

The association between the distribution of CMBs (deep or lobar) and cognitive impairment has been reported, but the results are controversial^19–23^. Some studies have reported that deep CMBs, particularly those in the basal ganglia, are associated with cognitive impairment^19,20^. whereas others have demonstrated that lobar CMBs are associated with cognitive impairment^21–23^, which supports our results. Chung et al. reported that in a community-based study in Taiwan, lobar CMBs were associated with cognitive impairment^22^, which is consistent with our results. In addition, van Norden et al. reported that among nondemented patients who had SVDs, lobar CMBs were associated with cognitive impairment, which is similar to our results. Several reasons for the controversy are considered. There are differences in the target subjects. Yakushiji et al. reported that among Japanese adults without neurological disorders who received brain health screening tests, deep CMBs were associated with cognitive impairment^19^. In Japan, brain health screening tests with MRI are commonly performed. These screening tests were performed based on requests, and thus, potential bias exists. In particular, because almost all healthy subjects received brain health screening tests, fewer subjects had CMBs than that in the general population. In fact, according to this report, the number of cognitive subnormal subjects with lobar CMBs was only 2 among 1279 subjects. Different study designs might show the opposite result. However, the distribution of CMBs is different between eastern and western countries^33^. The prevalence of deep or mixed CMBs is higher in eastern countries than in western countries. In this study, among 73 patients with CMBs, 61 (83.6%) patients had deep or mixed CMBs, which was consistent with the findings of a previous report^33^. However, the mechanism and pathological meanings were similar regardless of the difference in distribution.

The pathological differences in CMBs according to distribution are well known, with CMBs in deep regions believed to be associated with hypertensive microangiopathy, whereas lobar MBs share risk factors with CAA^11,12^. Because the locations of the CMBs may reflect different mechanisms^34,35^, distinct underlying pathologies may mediate the relationship between CMBs and cognitive impairment. CAA is characterized by a progressive deposition of amyloid-β in the media and adventitia of the arterioles, capillaries, and venules in the cerebral cortex and the subcortex. The relationship between lobar CMBs and cognitive decline in this study implies that CAA might be an underlying pathology in CMB-related cognitive impairment. Because CAA pathology is frequently observed in AD^36,37^, the relationship between CAA, apart from AD pathology, and cognitive impairment has been discussed. A recent autopsy-based study reported that CAA pathology is associated with an increased rate of cognitive impairment^37^. These associations were independent of AD pathology, which supports a role for CAA as an important and independent contributor to cognitive impairment. In this study, the subjects were patients with a first-ever lacunar infarction, in whom cSVDs, especially hypertensive microangiopathy, might have progressed to some extent. However, lobar CAA contributed to cognitive impairment. Moreover, the MMSE score was lower in the CMB-lobar group than in the CMB-mixed group, which suggested that lobar CMBs originating from CAA independently promote the pathological process and strongly influence cognitive impairment.

In this investigation, the subscores for orientation and visuospatial cognition were lower in the CMB-lobar group than the CMB-negative group. However, the mechanisms underlying the associations between the pathological and physiological mechanisms remain unclear. Deep CMBs were reported to be associated with the impairment of attention and calculation^19^. In this investigation, the subscores for attention and calculation were relatively low in all of the patients who had lacunar infarcts. These findings suggested that hypertensive microangiopathy but not deep CMBs might contribute to the impairment of attention and calculation. However, further studies on the correlations between neuroimaging and pathology are needed to validate the specificity of the relationship with CAA-related cognitive impairment.

This study had several limitations. First, sampling bias might exist. This study was a single-center retrospective study. First-ever lacunar infarction patients were included, and dementia patients were excluded. In this study, we focused on cSVDs and searched for factors with an impact on cognitive impairment. Therefore, we excluded dementia patients because such patients frequently have neurodegenerative pathologies such as tauopathy and synucleinopathy. As shown in previous studies, the target subjects strongly influenced the results and conclusions. The sample size and possible selection bias were considered. Study designs should be adequately discussed. Second, in this study, cognitive assessment was performed only with the MMSE. Other tests, such as the Montreal cognitive assessment, could detect slight cognitive impairment. Various cognitive assessment indexes should be used. Third, poststroke depression and apathy may influence the MMSE score. In this study, we did not evaluate these factors. According to a meta-analysis, poststroke depression in the acute stroke phase is significantly associated with frontal and basal ganglia lesions, and poststroke apathy is related to hemorrhagic stroke^38^. In this study, we only included patients with first-ever acute stroke diagnosed with lacunar infarction. We evaluated the association of stroke lesions with the MMSE score and could not find any association. No patients had hemorrhagic stroke in this study cohort. Therefore, the associations of poststroke depression and apathy with the MMSE score may be limited in this study.

In conclusion, for patients with a first-ever lacunar infarction, lobar CMBs were strongly associated with cognitive impairment. CAA is an important factor in cognitive impairment.

## Supplementary information

Supplementary table1

Supplementary table2

## Data Availability

The data that support the findings of this study are available from the corresponding author on reasonable request.
