# Supplementary material for: Lobar microbleeds are associated with cognitive impairment in patients with lacunar infarction
Source: Sci Rep. 2020 Oct 2;10:16410. doi: 10.1038/s41598-020-73404-6 (PMC7532194; doi:10.1038/s41598-020-73404-6)
Supplement: Supplementary file 1 — Supplementary table1 [file 41598_2020_73404_MOESM1_ESM.docx]

**Supplemental Table 1 Comparison of patient backgrounds between the lobar CMB-positive and lobar CMB-negative groups**

|  | Lobar CMB-positive (n=36) | Lobar CMB-negative (n=237) | p-value |
| --- | --- | --- | --- |
| Age, year | 73.7±9.0 | 71.8±11.4 | 0.347 |
| Sex (female), n (%) | 8 (22.2) | 87 (36.7) | 0.089 |
| Body mass index, kg/m^2^ | 23.6±3.1 | 23.8±3.6 | 0.803 |
| Education, year | 11.8±2.4 | 12.4±2.4 | 0.210 |
| MMSE score, median (IQR) | 25.5 (22–28.75) | 27 (26–29) | <0.001* |
| Hypertension, n (%) | 30 (83.3) | 176 (74.3) | 0.239 |
| Diabetes mellitus, n (%) | 6 (16.7) | 63 (26.6) | 0.202 |
| Dyslipidemia, n (%) | 16 (44.4) | 135 (57.0) | 0.159 |
| Chronic kidney disease, n (%) | 17 (47.2) | 65 (27.4) | 0.016* |
| Current smoker, n (%) | 14 (38.9) | 88 (37.1) | 0.839 |
| Habitual drinker, n (%) | 15 (42.9) | 94 (39.7) | 0.719 |

CMBs, cerebral microbleeds; MMSE, Mini-Mental Scale Examination; IQR, interquartile range. Data are presented as the mean±standard deviation, median (25% IQR–75% IQR), or number of patients (%).* indicates <0.05
