# Supplementary material for: Lobar microbleeds are associated with cognitive impairment in patients with lacunar infarction
Source: Sci Rep. 2020 Oct 2;10:16410. doi: 10.1038/s41598-020-73404-6 (PMC7532194; doi:10.1038/s41598-020-73404-6)
Supplement: Supplementary file 2 — Supplementary table2 [file 41598_2020_73404_MOESM2_ESM.docx]

**Supplemental Table 2 Associations between multiple factors (including the number of CMBs) and decreases in MMSE score**

|  | Univariate analysis | Multivariate analysis | | |
| --- | --- | --- | --- | --- |
|  | p value | Predictive value | p-value | VIF |
| Age | <0.001 | -0.058 | 0.007 | 2.83 |
| Sex (female) | 0.392 |  |  |  |
| Body mass index | 0.001 | 0.077 | 0.067 | 1.13 |
| Education | 0.001 | 0.002 | 0.981 | 1.44 |
| Hypertension | 0.353 |  |  |  |
| Diabetes mellitus | 0.214 |  |  |  |
| Dyslipidemia | 0.113 | 0.036 | 0.808 | 1.10 |
| Chronic kidney disease | 0.002 | -0.275 | 0.085 | 1.09 |
| Current smoker | 0.494 |  |  |  |
| Habitual drinker | 0.221 |  |  |  |
| Location of infarction |  |  |  |  |
| corona radiata | 0.826 |  |  |  |
| basal ganglia | 0.608 |  |  |  |
| capsulae internae | 0.718 |  |  |  |
| thalamus | 0.259 |  |  |  |
| brain stem | 0.335 |  |  |  |
| MRI findings |  |  |  |  |
| DWMH severe | 0.304 |  |  |  |
| PVH severe | 0.056 | -0.010 | 0.951 | 1.19 |
| Medial temporal atrophy | <0.001 | -1.444 | <0.001 | 2.81 |
| Number of deep CMBs | 0.001 | -0.030 | 0.363 | 2.85 |
| Number of lobar CMBs | <0.001 | -0.208 | 0.001 | 2.82 |

CMBs, cerebral microbleeds; MMSE, Mini-Mental Scale Examination; VIF, variance inflation factor; MRI, magnetic resonance imaging; DSWMH, deep and subcortical white matter hyperintensity; PVH, periventricular hyperintensity. * indicates <0.05
